# Telocinobufagin and Marinobufagin Produce Different Effects in LLC-PK1 Cells: A Case of Functional Selectivity of Bufadienolides

**DOI:** 10.3390/ijms19092769

**Published:** 2018-09-14

**Authors:** Luciana S. Amaral, Jainne Martins Ferreira, Danilo Predes, José Garcia Abreu, François Noël, Luis Eduardo M. Quintas

**Affiliations:** Institute of Biomedical Sciences, Federal University of Rio de Janeiro, Rio de Janeiro 21941-590, Brazil; lucianasamaral@yahoo.com.br (L.S.A.); jainne@gmail.com (J.M.F.); danilopredes@gmail.com (D.P.); garciajr@icb.ufrj.br (J.G.A.); fnoel@pharma.ufrj.br (F.N.)

**Keywords:** cardiotonic steroids, bufadienolides, Na^+^/K^+^-ATPase, functional selectivity, ERK1/2, GSK-3β, Wnt/β-catenin pathway

## Abstract

Bufadienolides are cardiotonic steroids (CTS) identified in mammals. Besides Na^+^/K^+^-ATPase inhibition, they activate signal transduction via protein–protein interactions. Diversity of endogenous bufadienolides and mechanisms of action may indicate the presence of functional selectivity and unique cellular outcomes. We evaluated whether the bufadienolides telocinobufagin and marinobufagin induce changes in proliferation or viability of pig kidney (LLC-PK1) cells and the mechanisms involved in these changes. In some experiments, ouabain was used as a positive control. CTS exhibited an inhibitory IC_50_ of 0.20 (telocinobufagin), 0.14 (ouabain), and 3.40 μM (marinobufagin) for pig kidney Na^+^/K^+^-ATPase activity and concentrations that barely inhibited it were tested in LLC-PK1 cells. CTS induced rapid ERK1/2 phosphorylation, but corresponding proliferative response was observed for marinobufagin and ouabain instead of telocinobufagin. Telocinobufagin increased Bax:Bcl-2 expression ratio, sub-G0 cell cycle phase and pyknotic nuclei, indicating apoptosis. Src and MEK1/2 inhibitors blunted marinobufagin but not telocinobufagin effect, which was also not mediated by p38, JNK1/2, and PI3K. However, BIO, a GSK-3β inhibitor, reduced proliferation and, as telocinobufagin, phosphorylated GSK-3β at inhibitory Ser9. Combination of both drugs resulted in synergistic antiproliferative effect. Wnt reporter activity assay showed that telocinobufagin impaired Wnt/β-catenin pathway by acting upstream to β-catenin stabilization. Our findings support that mammalian endogenous bufadienolides may exhibit functional selectivity.

## 1. Introduction

The Na^+^/K^+^-ATPase is an enzyme that actively transports ions Na^+^ and K^+^ across cell membranes and has cardiotonic steroids (CTS—cardenolides and bufadienolides) as specific inhibitors of its pumping function. More recently, Xie and coworkers have established that, besides performing the active ion transport, Na^+^/K^+^-ATPase also functions as a signal transducer through protein–protein interactions, relaying extracellular signals to intracellular compartments via the activation of cascades of different protein kinases, as well as production of second messengers [1,2]. This intracellular signaling is activated by binding of CTS to Na^+^/K^+^-ATPase using the Src tyrosine kinase as a transducer. Therefore, CTS could work as inhibitors of Na^+^/K^+^-ATPase activity and also as agonists via Na^+^/K^+^-ATPase/Src complex [3]. These signal pathways promote regulation of various cell functions such as hypertrophy and hyperplasia, apoptosis, contraction, and differentiation, which depends on the host signaling modules of each cell type [2,3]. Studies show that the Na^+^/K^+^-ATPase may interact with protein kinases, phosphatases, membrane transporters, and other cellular proteins, and these interactions make this enzyme an important signal transducer [3].

A number of CTS have been characterized as endogenous steroids in mammals, but their function is still controversial [4]. Physiological roles like sodium balance and blood pressure regulation and the involvement in pathological conditions such as hypertension, heart failure, chronic renal failure, cancer and psychiatric disorders have been postulated [4,5,6,7]. Moreover, new therapeutic uses of Na^+^/K^+^-ATPase ligands have been considered recently, for example, as new anticancer agents, less toxic drugs for heart failure, and new substances against hypertension, cystic fibrosis, ischemic stroke, and neurodegenerative disorders [8,9]. The antitumoral effect of some CTS in vitro and in vivo has led to a growing interest in the development of CTS for cancer [10,11,12]. Interestingly, CTS usually have different effects in normal cells compared to tumor cells, so they can be inactive or proliferative to the former while kills the latter [13,14]. Thus, even though the signaling pathways are similar, the outcome seems to be distinct. On the other hand, few works have suggested that, in the same system, different effects may be elicited by specific CTS. For instance, ouabain attenuates the cardiotoxicity induced by other CTS like digoxin and bufalin [15]. On the other hand, digoxin blocks the hypertensinogenic effect of ouabain, maybe because only ouabain activates Src in vascular smooth myocytes [16,17]. In Madin-Darby Canine Kidney (MDCK) cells from distal tubule, cytotoxicity triggered by several CTS like ouabain and bufalin is not shared by marinobufagin and marinobufotoxin [18]. In isolated rat kidneys, we demonstrated that, at equipotent concentrations for Na^+^/K^+^-ATPase inhibition, bufalin stimulates natriuresis and diuresis but not ouabain [19].

Recently, some studies with G protein-coupled receptors (GPCR) have shown that ligands produce different qualitative effects even when interacting with the same and single receptor. One explanation is that different conformational states of a receptor would be induced or selected by ligands. Thus, they could differentially activate signaling pathways mediated by the same receptor and act as both agonists and antagonists of different functional cascades [20,21,22]. This mechanism, known as functional selectivity/biased agonism, is a relatively novel concept in pharmacology, and may explain why several similar endogenous ligands are produced by the organism, and also raises the possibility of selecting or designing novel ligands that differentially activate only a subset of functions of a single receptor, thereby optimizing therapeutic action. In this work we compared the effect of two chemically similar bufadienolides, telocinobufagin, and marinobufagin, on the inhibition of Na^+^/K^+^-ATPase with their cellular effects and the molecular mechanisms involved, and our results suggest that they have functional selectivity.

## 2. Results

### 2.1. Inhibition of Pig Kidney Na^+^/K^+^-ATPase Activity

**Figure 1:**
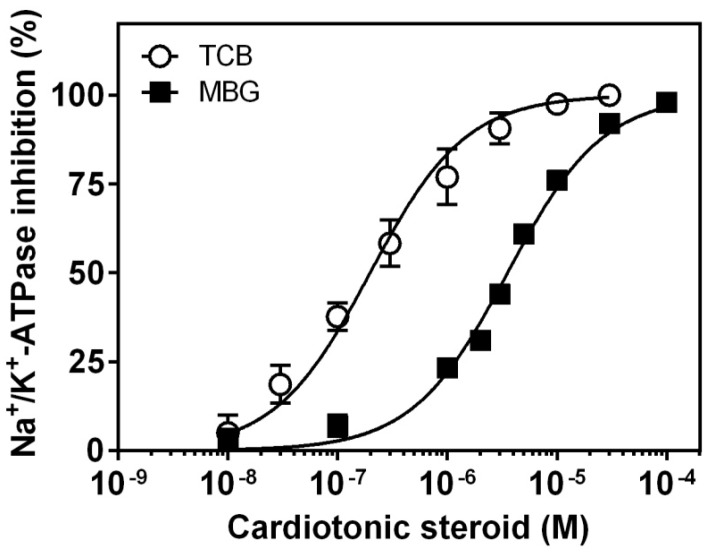
Inhibition curves of pig kidney Na^+^/K^+^-ATPase activity by telocinobufagin (TCB) or marinobufagin (MBG). Preparation of Na^+^/K^+^-ATPase from pig kidney was incubated with increasing concentrations of TCB or MBG for 1 h. Each point represents the mean ± SEM of three independent experiments performed in triplicate.

The potency of bufadienolides for inhibiting Na^+^/K^+^-ATPase activity is shown in Figure 1. Both bufadienolides fully inhibited enzyme activity, but telocinobufagin was around 15 times more potent than marinobufagin (IC_50_ = 0.20 ± 0.02 and 3.40 ± 0.18 μM, respectively). As a comparison, the IC_50_ of ouabain, a well-known CTS, was 0.14 ± 0.02 μM, similar to telocinobufagin (Figure S1).

### 2.2. Effect of Bufadienolides on ERK1/2 Activation

**Figure 2:**
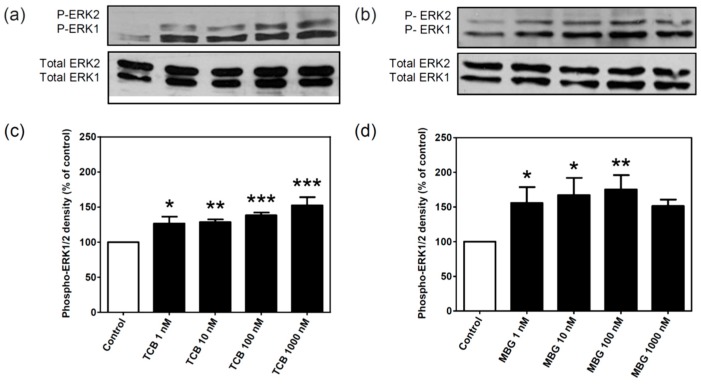
ERK1/2 activation by different concentrations of telocinobufagin (TCB) or marinobufagin (MBG). Serum-starved LLC-PK1 cells were treated with 1, 10, 100, and 1000 nM TCB or MBG for 15 min. Representative Western blots of phospho- and total ERK1/2 for TCB (**a**) and MBG (**b**) and relative optical density quantification in (**c**,**d**), respectively. Data are mean ± SEM of five independent experiments. * *p* < 0.05; ** *p* < 0.01; *** *p* < 0.005 vs. control.

CTS, especially ouabain, have been shown to induce prompt mitogen-activated protein kinases ERK1/2 activation at low concentrations [23]. Figure 2 shows that both telocinobufagin (Figure 2a,c) and marinobufagin (Figure 2b,d) in the nanomolar range stimulated ERK1/2 phosphorylation after 15 min incubation at concentrations that barely inhibit Na^+^/K^+^-ATPase activity. Ouabain was used as a positive control (Figure S2).

### 2.3. Effect of Bufadienolides on Cell Proliferation and Viability

**Figure 3:**
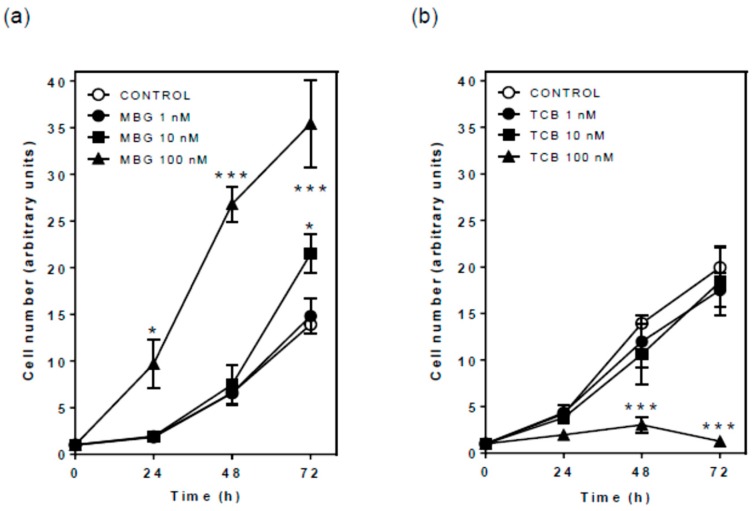
Cell proliferation of LLC-PK1 cells treated with marinobufagin (MBG) or telocinobufagin (TCB). Serum-starved LLC-PK1 cells were treated with 1, 10, and 100 nM MBG (**a**) or TCB (**b**) in 2.5% FBS for 24, 48, and 72 h, and then Trypan blue-free viable cells were counted in Neubauer chamber. Each point represents the mean ± SEM of three independent experiments performed in duplicate. * *p* < 0.05; *** *p* < 0.005 vs. control.

ERK pathway is associated with various cellular functions such as growth and CTS like ouabain and marinobufagin have been described to stimulate proliferation of normal cells [14,24,25]. Cell counting with Trypan blue exclusion up to 72 h demonstrated that marinobufagin, similar to ouabain (Figure S3), promoted significant cell growth after 72 h at 10 nM, and 24, 48, and 72 h at 100 nM (Figure 3a). On the contrary, telocinobufagin did not affect cell proliferation at 1 and 10 nM, and, in contrast to the other CTS, significantly hampered cell growth after 48 h at 100 nM (Figure 3b), with rare cells stained with Trypan blue dye. 

**Figure 4:**
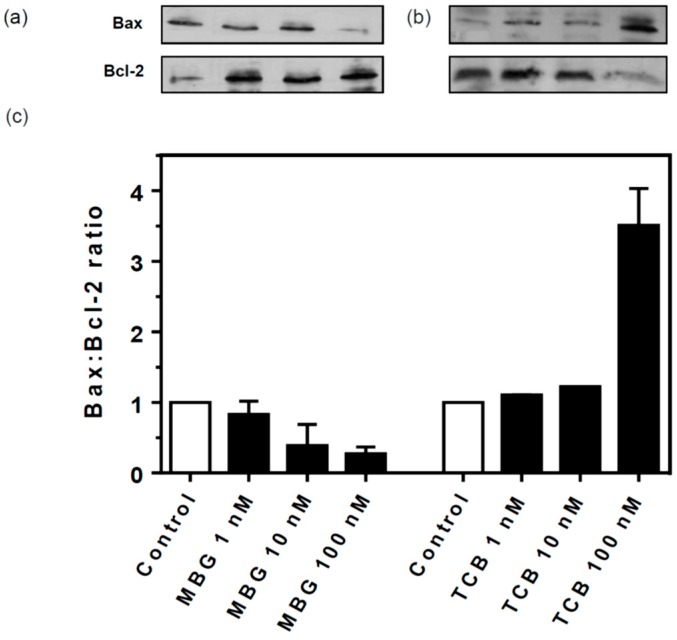
Bax and Bcl-2 expression in LLC-PK1 cells treated with marinobufagin (MBG) and telocinobufagin (TCB). Serum-starved LLC-PK1 cells were treated with 1, 10, and 100 nM MBG and TCB in 2.5% FBS for 72 h. Representative western blots of the pro-apoptotic Bax and anti-apoptotic Bcl-2 for MBG (**a**) and TCB (**b**) and the ratio of the relative optical density quantification for Bax:Bcl-2 (**c**). Data are the mean ± SEM of two independent experiments.

To investigate in more detail the effects found on cell proliferation, we decided to test the effects of bufadienolides on the expression of markers of cell viability, the anti-apoptotic protein Bcl-2 and the pro-apoptotic protein Bax in LLC-PK1 cells treated for 72 h. Consistently, whether Bax expression decreased with marinobufagin, Bcl-2 expression increased, similar to ouabain (Figure S4); the contrary was observed with telocinobufagin (Figure 4a,b, respectively). Figure 4c shows the densitometric analysis consistent with a decrease of Bax:Bcl-2 ratio in marinobufagin-treated cells, explaining the increase in proliferation, but an increase in telocinobufagin-treated cells, suggesting the onset of apoptosis.

### 2.4. Effect of Telocinobufagin on Cell Cycle Phases and Cell Death

**Figure 5:**
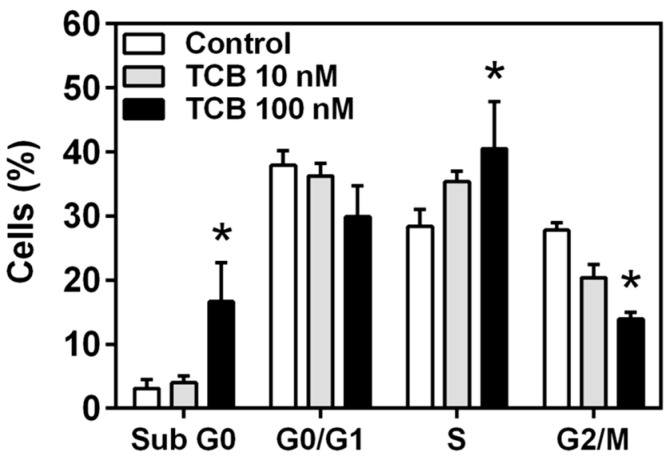
Cell cycle analysis of LLC-PK1 cells treated with telocinobufagin (TCB) by flow cytometry. Serum-starved LLC-PK1 cells were treated with 10 and 100 nM TCB in 2.5% FBS for 48 h. Distribution of cells in the sub G0, G0/G1, S and G2/M phases of the cell cycle. Data are the mean ± SEM of three independent experiments in duplicate. * *p* < 0.05 vs. control.

**Figure 6:**
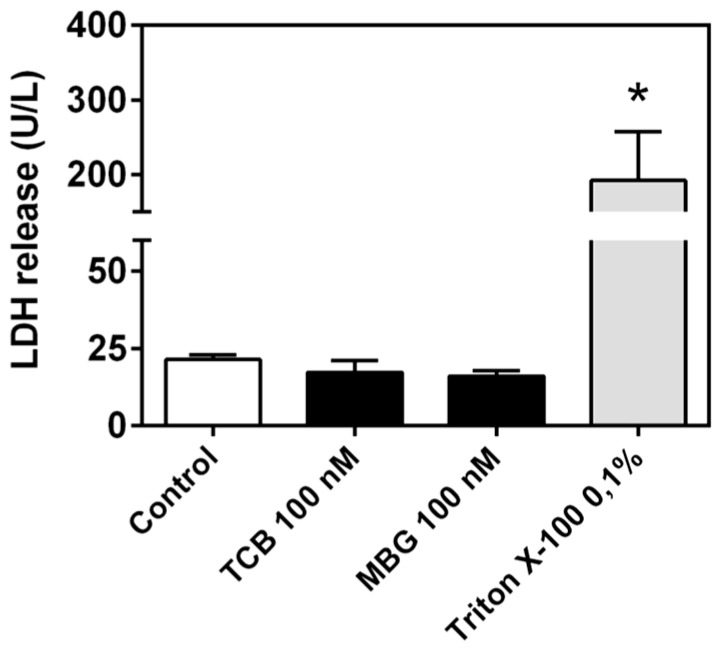
Lactate dehydrogenase (LDH) release from LLC-PK1 cells treated with telocinobufagin (TCB) or marinobufagin (MBG). Serum-starved LLC-PK1 cells were treated with 100 nM TCB or 100 nM MBG in 2.5% FBS for 72 h. Data are the mean ± SEM of three independent experiments in duplicate. * *p* < 0.05 vs. control. Triton X-100 was used as positive control.

Since 100 nM telocinobufagin had an antiproliferative effect and reduced cell viability, we decided to evaluate alterations in the phases of the cell cycle through flow cytometry. At 48 h, only 100 nM telocinobufagin significantly changed cell cycle phase profile, promoting a 5.5-fold increase of cells in sub-G0 and 1.5-fold in S phase and a 50% decrease of cells in G2/M phase (Figure 5). Along with these results, LDH release, a marker of necrotic cell death, was not different from control for both bufadienolides (Figure 6).

**Figure 7:**
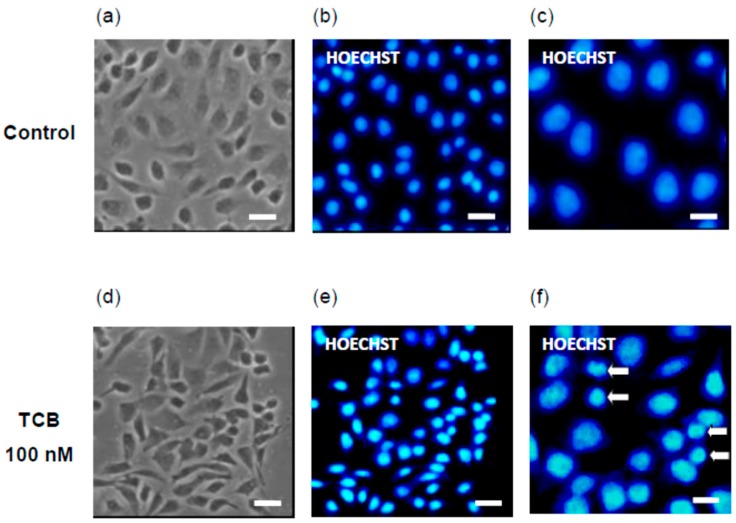
Apoptotic morphology of LLC-PK1 cells treated with telocinobufagin (TCB). Serum-starved LLC-PK1 cells were treated with 100 nM TCB in 2.5% FBS for 24 h. Phase-contrast microscopy (**a**: control; **d**: TCB) and Hoechst 33,342 staining for nuclear fluorescence (**b**,**c**: control; **e**,**f**, TCB). Higher magnification is shown in (**c**,**f**). Arrows indicate cells with pyknotic nuclei in (**f**). Scale bar: 50 μm (**a**,**b**,**d**,**e**); 20 μm (**c**,**f**).

Hoechst staining was used to observe nuclear morphological alterations induced by telocinobufagin. Figure 7 shows that, compared to control (Figure 7b,c), condensation of nuclear chromatin, seen as smaller and brighter nuclei, can be detected in 100 nM telocinobufagin-treated cells (Figure 7e,f), corroborating that apoptosis is induced by such bufadienolide at this concentration.

### 2.5. Role of Intracellular Ca^2+^ on Telocinobufagin-Induced Cell Death

**Figure 8:**
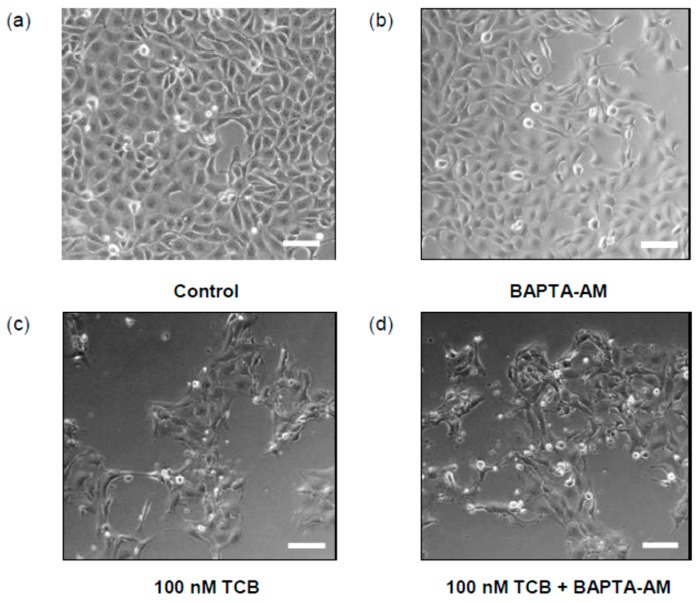
Effect of BAPTA-AM on morphology of LLC-PK1 cells treated with telocinobufagin (TCB). Serum-starved LLC-PK1 cells were treated with 100 nM TCB and/or 5 μM BAPTA-AM in 2.5% FBS for 24 h and phase-contrast microscopy was performed for control (**a**), BAPTA (**b**), and TCB-treated cells without (**c**) or with BAPTA (**d**). Scale bar: 100 μm.

CTS are classical inhibitors of Na^+^/K^+^-ATPase which may increase intracellular [Ca^2+^]. In LLC-PK1 cells, 100 nM ouabain was shown to produce Ca^2+^ transients [26] and this ion is involved in cell death [27]. We used BAPTA-AM, an intracellular Ca^2+^ chelator, in order to evaluate the association of Ca^2+^ and telocinobufagin effect. After pre-incubation with 5 μM BAPTA-AM, cells were treated with 100 nM telocinobufagin for 24 h. As exhibited in Figure 8, BAPTA-AM could not prevent the effect of telocinobufagin.

### 2.6. Role of Caveolae on Telocinobufagin-Induced Cell Death

**Figure 9:**
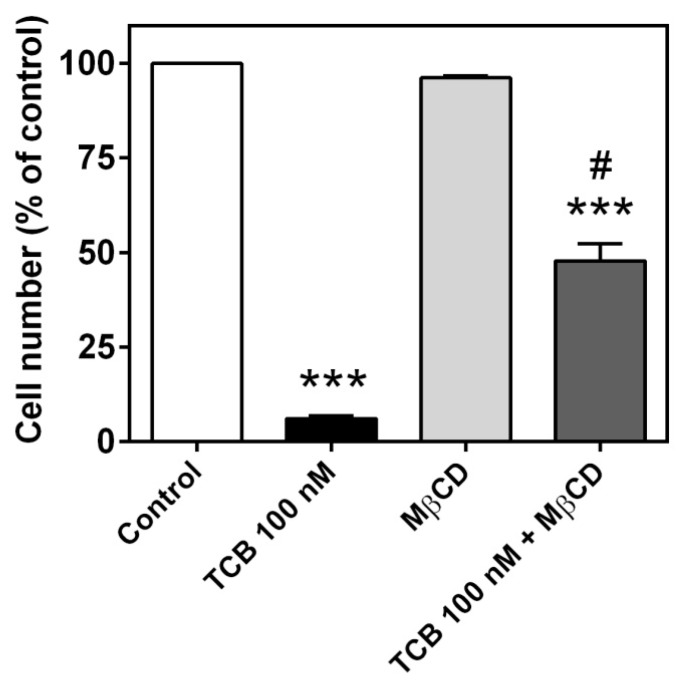
Effect of MβCD on cell proliferation of LLC-PK1 cells treated with telocinobufagin (TCB). Serum-starved LLC-PK1 cells were treated with 100 nM TCB and/or pretreated with 10 mM MβCD for 30 min and then with 1 mM MβCD in 2.5% FBS for 72 h and Trypan blue-free viable cells were counted in Neubauer chamber. Data are the mean ± SEM of three independent experiments in duplicate. *** *p* < 0.005 vs. control. # *p* < 0.005 vs. TCB.

The Na^+^/K^+^-ATPase population that mediates signal transduction has been shown to reside in caveolae, which are invaginations of the plasma membrane rich in cholesterol [23,28,29]. Methyl-β-cyclodextrin (MβCD) has been used to hamper Na^+^/K^+^-ATPase-mediated signaling by depleting cholesterol and thus disrupting caveolae [30,31,32]. After a 30-min pretreatment with 10 mM MβCD, LLC-PK1 cells were incubated with 1 mM MβCD plus 100 nM telocinobufagin for 72 h. As shown in Figure 9, such treatment partially prevented telocinobufagin effect.

### 2.7. Signaling Pathways Involved in Bufadienolide Effect

**Figure 10:**
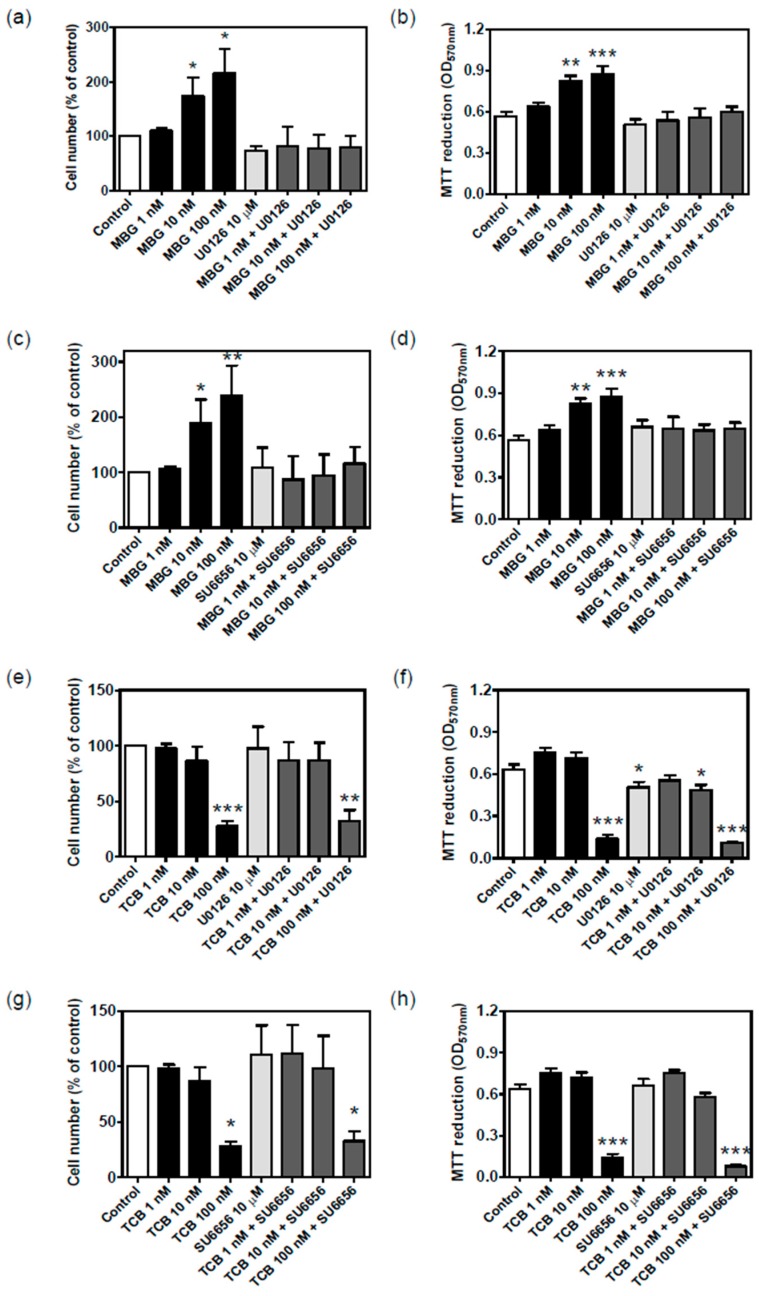
Effect of MEK1/2 and Src inhibition on cell proliferation and viability of LLC-PK1 cells treated with marinobufagin (MBG) and telocinobufagin (TCB). Serum-starved LLC-PK1 cells were treated with 1, 10, and 100 nM MBG (**a**–**d**) and TCB (**e**–**h**) in 2.5% FBS for 72 h with or without 10 μM U0126 (MEK inhibitor) (**a**,**b**,**e**,**f**) or 10 μM SU6656 (Src inhibitor) (**c**,**d**,**g**,**h**). Trypan blue-free viable cells were counted in Neubauer chamber (**a**,**c**,**e**,**g**) and viability was assessed by MTT (**b**,**d**,**f**,**h**). Data are the mean ± SEM of at least three independent experiments in duplicate. * *p* < 0.05; ** *p* < 0.01; *** *p* < 0.005 vs. control.

In order to check whether ERK1/2 pathway is linked to the cellular effect of bufadienolides, we used the inhibitor of the upstream kinases MEK1/2 and Src, two classical sequential points in the signaling pathway mediated by Na^+^/K^+^-ATPase. Marinobufagin 10 and 100 nM stimulated cell proliferation at 72 h. The treatment with MEK inhibitor (Figure 10a,b) and Src inhibitor (Figure 10c,d) prevented this effect in both proliferation (Figure 10a,c) and viability assays (Figure 10b,d). Conversely, the antiproliferative/cell death effect of 100 nM telocinobufagin was not prevented by such inhibitors (Figure 10e–h).

**Figure 11:**
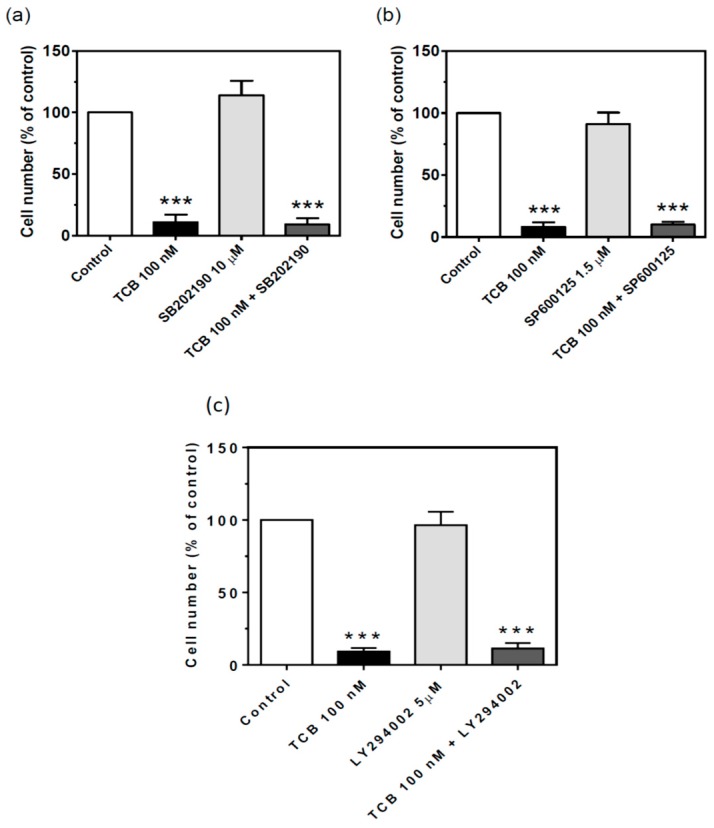
Effect of p38, JNK1/2 and PI3K inhibition on cell proliferation of LLC-PK1 cells treated with telocinobufagin (TCB). Serum-starved LLC-PK1 cells were treated with 100 nM TCB in 2.5% FBS for 72 h with or without 10 μM SB202180 (p38 inhibitor) (**a**), 1.5 μM SB600125 (JNK1/2 inhibitor) (**b**), or 5 μM LY294002 (PI3K inhibitor) (**c**). Trypan blue-free viable cells were counted in Neubauer chamber. Data are the mean ± SEM of at least three independent experiments in duplicate. *** *p* < 0.005 vs. control.

CTS-evoked Na^+^/K^+^-ATPase signaling has been shown to activate stress-activated kinases p38 [33] and JNK1/2 [34] and may induce cell death. To test the involvement of these kinases in the effect of telocinobufagin, we treated our LLC-PK1 cells with the p38 inhibitor SB202190 (10 μM) and JNK inhibitor SP600125 (1.5 μM). Figure 11 shows that these treatments but did not prevent the effect of telocinobufagin. Inhibition of PI3K/AKT/mTOR pathway is related to the antiproliferative effect of some CTS [35,36,37]. Incubation of LLC-PK1 cells with the PI3K inhibitor LY2940025 (5 μM) alone did not affect the number of viable cells and also had no effect on telocinobufagin-treated cells (Figure 11c).

**Figure 12:**
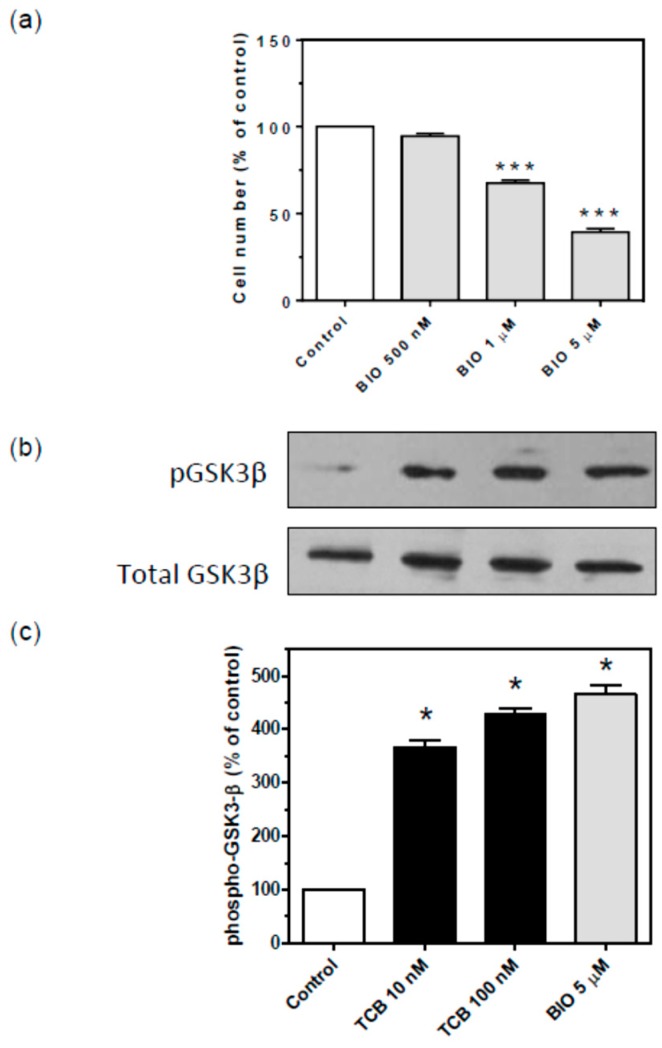
Effect of GSK-3β inhibition on cell proliferation and GSK-3β Ser9 phosphorylation of LLC-PK1 cells. Serum-starved LLC-PK1 cells were treated with 0.5, 1, and 5 μM BIO (GSK-3β inhibitor) in 2.5% FBS for 72 h (**a**), and 10 and 100 nM TCB and 5 μM BIO for 15 min (**b**,**c**). Trypan blue-free viable cells were counted in Neubauer chamber (**a**). Representative western blots of phospho- and total GSK-3β (**b**) and relative optical density quantification in (**c**). Data are the mean ± SEM of at least three independent experiments. * *p* < 0.05; *** *p* < 0.005 vs. control.

**Figure 13:**
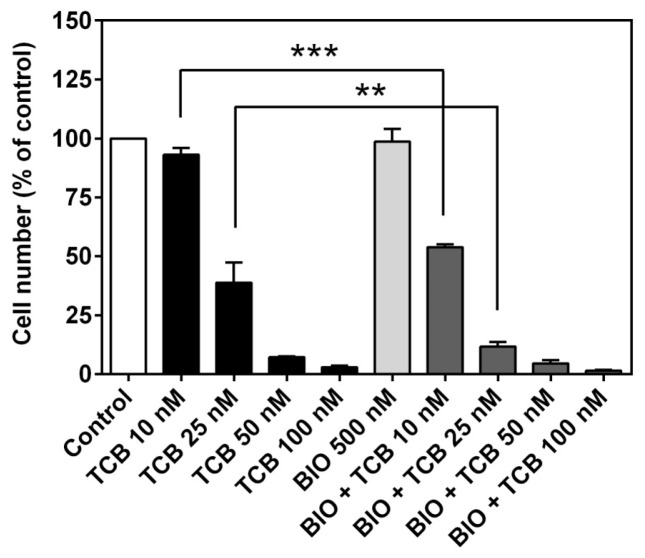
Effect of GSK-3β inhibition on cell proliferation of LLC-PK1 cells treated with telocinobufagin (TCB). Serum-starved LLC-PK1 cells were treated with 10, 25, 50 and 100 nM TCB in 2.5% FBS for 72 h with or without 500 nM BIO (GSK-3β inhibitor). Trypan blue-free viable cells were counted in Neubauer chamber. Data are the mean ± SEM of at least three independent experiments in duplicate. ** *p* < 0.01 vs. TCB 25 nM; *** *p* < 0.005 vs. TCB 10 nM.

As GSK-3β is a constitutively active kinase critical for cellular functions including proliferation and repression of epithelial-mesenchymal transition (EMT) [38] and its modulation has been ascribed as an apoptotic mechanism of CTS [39], we also tested this pathway. First, we evaluated the effect of the GSK-3β inhibitor 6-bromoindirubin-3′-oxime (BIO) on LLC-PK1 cell number. A concentration-dependent inhibitory effect was observed (Figure 12a). Second, we showed that 10 and 100 nM telocinobufagin promoted an inhibitory GSK-3β phosphorylation at Ser9, comparable to 5 μM BIO (Figure 12b,c). Then, we evaluated the combination effect of 500 nM BIO, which was not able to reduce cell growth when used alone, in the presence of different concentrations of telocinobufagin. As shown in Figure 13, while BIO had no effect, it potentiated telocinobufagin at concentrations of 10 and 25 nM.

**Figure 14:**
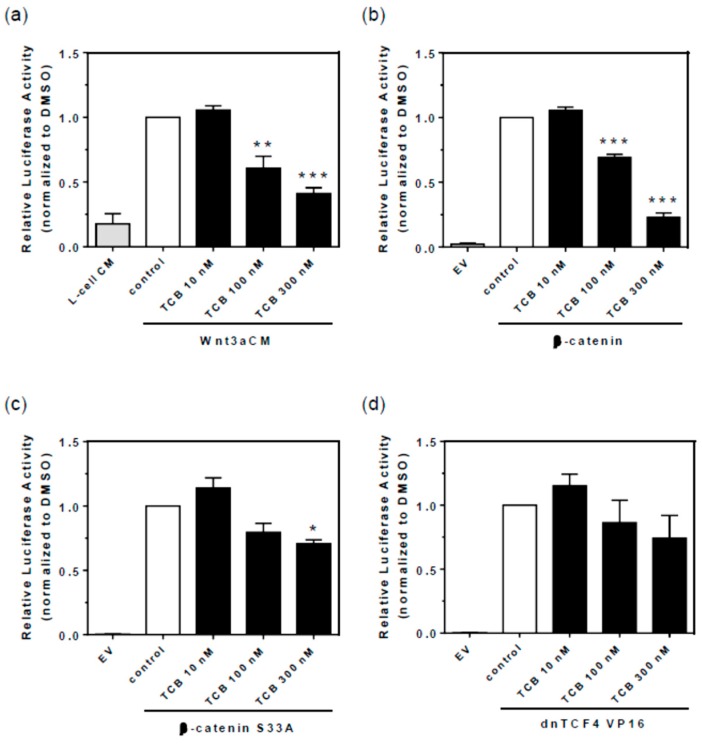
Wnt/β-catenin signaling inhibition by TCB relies on β-catenin phosphorylation and degradation. Wnt/β-catenin signaling pathway specific TOPFLASH gene reporter assay for LLC-PK1 cells treated with 10, 100, and 300 nM TCB with control L-cell (L-cell CM) or Wnt3a conditioned medium (Wnt3a CM) for inducing Wnt/β-catenin pathway for 24 h (**a**). Cells were transfected with β-catenin, a constitutively active β-catenin mutant (**b**), β-catenin S33A (**c**), or constitutively active TCF4 construct dnTCF4 VP16 (**d**). Data are the mean ± SEM of at least three independent experiments. * *p* < 0.05; ** *p* < 0.01; *** *p* < 0.005 vs. control. EV, empty vector.

GSK-3β is particularly involved in EMT, being one of the checkpoints of Wnt/β-catenin pathway. When active, it impairs Wnt signaling by phosphorylation of β-catenin leading to proteasomal degradation. The inhibitory effect of telocinobufagin on GSK-3β would allow β-catenin translocation to the nucleus and development of EMT. These data suggest that telocinobufagin could activate Wnt/β-catenin signaling through GSK-3β inhibition. In order to assay how telocinobufagin could activate or inhibit Wnt/β-catenin signaling, we activated the Wnt/β-catenin pathway at four different levels and checked whether telocinobufagin could modulate Wnt/β-catenin pathway activation. We treated LLC-PK1 cells with Wnt3a conditioned medium (Wnt3a CM), or the control medium L-cell conditioned medium (L-cell CM), in order to access the inhibition of the physiological Wnt signaling activity. We also transfected LLC-PK1 cells with either β-catenin, β-catenin S33A (a constitutively active mutant of β-catenin, that cannot be phosphorylated by the destruction complex and thus is not degraded), or dnTCF4 VP16 (a constitutively active form of TCF4 that does not rely on β-catenin to activate the signaling). Telocinobufagin treatment inhibited 40% at 100 nM and 59% at 300 nM the TOPFLASH reporter activity of cells treated with Wnt3a CM (Figure 14a) and 31% at 100 nM and 77% at 300 nM the activity of β-catenin transfected cells (Figure 14b). The TOPFLASH reporter activity of the β-catenin S33A transfected cells was inhibited only 30% at 300 nM telocinobufagin, and not at 100 nM (Figure 14c). TCB treatment displayed no effect upon TOPFLASH reporter activity of dnTCF4 VP16 transfected cells (Figure 14d). These data strongly suggest that telocinobufagin inhibits Wnt/β-catenin signaling downstream to β-catenin stabilization and that telocinobufagin activity relies on β-catenin phosphorylation and degradation. Hence, telocinobufagin acts at two different levels on Wnt/β-catenin signaling pathway. While it inhibits GSK-3β, it also impairs β-catenin stabilization by an undescribed mechanism.

## 3. Discussion

Several CTS have been found in mammals and their binding to Na^+^/K^+^-ATPase have been considered to exert similar effects by the same mechanism of action. The discovery of novel roles of Na^+^/K^+^-ATPase has gradually changed the consensus view of CTS physiopharmacology. For the present work we chose two bufadienolides discovered in human plasma, telocinobufagin and marinobufagin, with strikingly similar chemical structure, and showed that they produced different pharmacological effects in the same cell, suggesting that CTS can exhibit functional selectivity.

CTS are classical ligands of Na^+^/K^+^-ATPase and bind with different potencies depending on the isozyme and animal species [40]. In contrast to most mammalian cells, renal epithelial cells basically express one isozyme in abundance, α1β1, and serve as a good model to evaluate the effect of CTS without the interference of other isozymes. Moreover, pig kidney α1β1 is sensitive to CTS and has been widely used to dissect binding and Na^+^/K^+^-ATPase-mediated signaling properties of these compounds and is an appropriate model of human (kidney) Na^+^/K^+^-ATPase [41]. We first evaluated the inhibitory potency of telocinobufagin and marinobufagin on the enzymatic activity of pig kidney Na^+^/K^+^-ATPase. Telocinobufagin had an IC_50_ around 200 nM, equivalent to ouabain which was used as a reference CTS. On the other hand, although marinobufagin differs from telocinobufagin only by the presence of an epoxide group at C14–C15 instead of a hydroxyl group at C14 in the steroidal nucleus, the former was much less potent (IC_50_ around 3400 nM). These results are consistent with our previous findings in rat [42] and human kidney [43].

In addition to Na^+^/K^+^-ATPase inhibition, CTS are now known to stimulate Na^+^/K^+^-ATPase-mediated intracellular signaling pathways by protein–protein interaction independent on its ion pumping function. This has been discovered by Zijian Xie and coworkers in the late 1990s and revolutionized the field with a series of groundbreaking studies (reviewed in, for instance, [3,44,45]). One of the first signaling pathways found to be mediated by Na^+^/K^+^-ATPase signalosome was the Src-dependent Na^+^/K^+^-ATPase-Ras-Raf-MEK1/2-ERK1/2, being this latter phosphorylated within minutes by CTS concentrations that hardly inhibit Na^+^/K^+^-ATPase. Indeed, 1–1000 nM telocinobufagin and marinobufagin stimulated ERK1/2 phosphorylation after 15 min. Ouabain, which has been well characterized as a Na^+^/K^+^-ATPase-mediated ERK1/2 activator in several cell types [46,47,48,49,50], including LLC-PK1 cells [51,52], also activated in the same concentration range. Reports on ERK1/2 activation by marinobufagin and telocinobufagin are scarce [42,53]. More important, most of the concentrations tested in the present work can barely inhibit Na^+^/K^+^-ATPase activity, which is in accordance to several works [54,55,56] and consistent with a nonpumping, signaling pool of Na^+^/K^+^-ATPase in LLC-PK1 cells [52]. 

The activation of ERK1/2 promotes cell viability and growth, and is a key mechanism responsible for CTS-induced cell proliferation [48,54,57]. Here, marinobufagin, as well as ouabain, induced cell proliferation at 10 and 100 nM, especially after 72 h. This effect has been reported for ouabain in LLC-PK1 [14] and other cell types [23,24,25,47,54,57,58]. Consistently, inhibition of Src and MEK1/2 completely hampered the proliferative effect of marinobufagin. Interestingly, the same effect was not shared by telocinobufagin, which did not affect cell proliferation at 10 nM compared to control and had a significant antiproliferative effect at 100 nM after 48 h. Note that it cannot be explained by different inhibitory potencies of telocinobufagin and marinobufagin since ouabain had the same cell proliferation profile as marinobufagin and exhibited a similar Na^+^/K^+^-ATPase inhibitory profile as telocinobufagin. Src and MEK1/2 blockade did not hamper TCB activity, indicating that another mechanism was involved.

The antiproliferative effect of CTS has been extensively studied in normal [25,59,60,61,62] and tumor cells, although the former cells usually are more resistant than the latter [9], resulting in repurposing of these compounds as promising anticancer drugs [10,39,63]. Our results of cell viability, increased Bax:Bcl-2 expression ratio, cell cycle analysis with a remarkable elevation of cells in sub-G0 (DNA fragmentation) phase and Hoechst 33342-stained cells with pyknotic nuclei reveal that apoptosis occurs in the presence of 100 nM telocinobufagin. Apoptosis is one of the main mechanisms responsible for CTS-evoked cell death [39,62]. Several molecular mechanisms have been considered for the apoptotic cell death induced by such compounds. At high concentrations, CTS block a significant fraction of Na+ pumps affecting ionic cell homeostasis, with the rise of intracellular Na^+^ concentration and ultimately Ca^2+^. This classical mechanism, described by Akera and Brody to explain the inotropic effect of CTS [64], has been also associated to CTS-induced apoptosis [13,55,62,65,66]. However, this is not the case here since the intracellular Ca^2+^ chelator BAPTA-AM did not influence telocinobufagin effect. Indeed, we showed that 100 nM telocinobufagin inhibited pig kidney Na^+^/K^+^-ATPase activity by 25%, probably insufficient for a significant change in the global intracellular Na^+^ concentration, as shown by Cai et al. for 100 nM ouabain in LLC-PK1 cells [67]. Moreover, stable LLC-PK1 cell lines knocked down for α1 are viable and do not exhibit significant morphological changes [68], demonstrating the large Na^+^ pump reserve capacity of these cells.

A complex array of different signaling pathways has been associated to cell growth arrest and death caused by CTS [69]. Caveolar Na^+^/K^+^-ATPase was revealed to be the signal transducer and strategies to dissipate caveolae also interrupt intracellular signaling [23,28,31]. We showed that MβCD can attenuate the effect of 100 nM telocinobufagin suggesting that this Na^+^/K^+^-ATPase pool has a role in triggering cell death. The inhibition of some putative second messengers responsible for CTS-induced cell death like p38 [33,70,71,72] and JNK1/2 [73,74,75] did not change telocinobufagin outcome. PI3K-Akt is a relevant pathway for LLC-PK1 cells viability and proliferation [14] and it was shown to be downstream ERK1/2 in proximal tubule renal cells treated with ouabain [47]. As for ERK1/2 (MEK1/2) inhibition, PI3K inhibition did not affect cell proliferation or telocinobufagin effect.

Another kinase, GSK-3β, mediates proliferation arrest and apoptosis [76]. Inhibition [39,47,77] or activation [37,78,79,80] of GSK-3β have been demonstrated to be induced by CTS. Our results showed that, unlike other tested kinase inhibitors, the GSK-3β inhibitor BIO decreased LLC-PK1 cell number in a concentration-dependent fashion and telocinobufagin, like BIO, stimulated GSK-3β phosphorylation at the deactivating site Ser9, suggesting a role of GSK-3β in telocinobufagin effect. When used in combination, BIO potentiated telocinobufagin-induced cell death. Because GSK-3β is a key regulator of Wnt/β-catenin pathway, and Wnt is secreted by kidney epithelial cell line [81,82], we chose this pathway to functionally substantiate the pGSK-3β Ser9 finding. In the resting state, GSK-3β is active and phosphorylates β-catenin triggering its degradation, antagonizing the canonical Wnt//β-catenin signaling. When Wnt binds to its receptor, GSK-3β is deactivated and β-catenin works as a transcription factor. Surprisingly, telocinobufagin inhibited, in contrast to stimulating, the Wnt signal. Telocinobufagin was able to inhibit β-catenin overexpression-induced Wnt signaling activation. Since the bufadienolide impairs Wnt signal transduction even with increased β-catenin levels, the inhibition of GSK-3β induced by telocinobufagin will not be sufficient to activate Wnt/β-catenin signaling. Moreover, Wnt/β-catenin activation through constitutively active β-catenin or dnTCF4 VP16 were not blocked by the bufadienolide, suggesting that telocinobufagin acts upstream to β-catenin stabilization. Recently, some CTS have been demonstrated to inhibit Wnt/β-catenin signaling in tumor cells [78,83,84] but, on the other hand, in renal cells, CTS have been reported to promote EMT [85,86] and β-catenin nuclear translocation [87]. Further studies are definitely necessary to characterize the mechanism(s) of action involved in telocinobufagin-induced LLC-PK1 cell death.

Functional selectivity hypothesis has been formulated in an attempt to explain phenomena that cannot be understood in the light of traditional pharmacological concepts. A central one is that pharmacological specificity occurs through interaction between the ligand and the receptor, where the ligands can be characterized by their receptor affinity and efficacy. Thus, the classification of agonist (full, partial, or inverse) and antagonist was created considering efficacy as a system-independent parameter, being constant for a particular ligand-receptor complex [88]. This dogma has been challenged in recent years, particularly by studies of G protein-coupled receptors, providing evidence that some ligands induce different responses mediated by the same and single receptor, depending on the system [21,22]. Therefore, the ligand would induce or select a thermodynamically favorable receptor conformation responsible to modulate the signal transduction and multiple possibilities of activation (or inactivation) of signaling pathways would be expected.

The debate concerning whether CTS have similar mechanism of action and pharmacological effect is not new. For instance, Runge et al. suggested that differences in CTS polarity were responsible for distinct cardiac effects [89], although pharmacokinetics was pointed out later to have a major role [90]. Pamnani et al. showed that bufalin, but not ouabain, produces a positive inotropic and chronotropic effect [91] and we demonstrated that bufalin is much more natriuretic and diuretic, despite of equivalent Na^+^/K^+^-ATPase inhibitory potency; this discrepancy occurs due to activation of Src-ERK1/2 pathway [19]. Ouabain induces hypertension when chronically administered in rats, while digoxin and digitoxin prevent this effect and can even reduce blood pressure when administered alone [16]. This may be related to the activation or not of Src-dependent signaling cascade [17]. Moreover, ouabain in low concentrations delays the cardiotoxic effect of digoxin and bufalin in vitro and in vivo, and apparently it seems to involve intracellular signaling mechanisms [15]. As the bufadienolides in our study are found endogenously in conditions such as hypertension, chronic renal failure, heart failure, and preeclampsia [4], an intriguing possibility is that the balance between them is important in health and disease. For instance, marinobufagin has been demonstrated to induce cardiac and renal fibrosis and it was consistent with the trigger of EMT in renal cells [85]. Perhaps, as for ouabain and digoxin in hypertension [16], the opposing effects of marinobufagin and telocinobufagin would maintain homeostasis and critical fluctuations of their endogenous concentration might be detrimental.

The molecular mechanism involved in the suggested functional selectivity of CTS is still elusive. Binding characteristics of distinct CTS may affect the Na^+^/K^+^-ATPase conformation. Laursen et al. showed by X-ray diffraction analysis of the crystal structure of ouabain-, digoxin-, and bufalin-(pig) α1β1 Na^+^/K^+^-ATPase complex that the size of the lactone ring, the substituents of the steroid nucleus as well as the degree of glycosylation are important to the depth of the CTS in the binding site and rearrangement of transmembrane segments [92]. On the other hand, Klimanova et al. showed that marinobufagin, different from ouabain that binds to E2-P conformation with high affinity (which is a consensus for CTS as a class), binds with similar affinity to E1 state duck α1β1 Na^+^/K^+^-ATPase [93]. A more complex situation was postulated by Song et al., who observed antagonism between “digoxin-like” and “ouabain-like” CTS [94], which in a certain way was also seen by Feldmann et al. [95] and Nesher et al. [16]. Although they exhibit an agonistic effect when tested alone in rat α2 and α3 Na^+^/K^+^-ATPase, they inhibit the effect of one another when combined. They proposed a model where Na^+^/K^+^-ATPase operates as tetramers (αβ)_4_ and the process of oligomerization and disaggregation of the oligomers would explain their findings [94].

## 4. Materials and Methods

### 4.1. Drugs

The bufadienolides telocinobufagin (m.w. 402.5 g/mol; CAS#: 472-26-4) and marinobufagin (m.w. 400.5 g/mol; CAS#: 470-42-8) were isolated by chromatographic separation on neutral aluminum oxide column from the parotoid gland secretion of the Brazilian toads *Rhinella schneideri* which were captured under the license of the Brazilian Institute for the Environment and Natural Resources/National Center for Research and Conservation of Reptiles and Amphibians (IBAMA/RAN) number 097/06 (process 02010.000832/04-74). Afterwards, the compounds were chemically characterized as described previously [96]. Briefly, the bufadienolides were structurally characterized by one- (^1^H and ^13^C) and two-dimensional—correlation spectroscopy, COSY; heteronuclear multiple-quantum (HMQC) and multiple-bond (HMBC) correlation—nuclear magnetic resonance (NMR) spectra and infrared (IR) experiments and are represented in Figure 1. Analytical data, Fourier transform IR data and chemical shifts for ^1^H and ^13^C NMR are presented in [96]. Stock solutions of bufadienolides (30 mM) were prepared in DMSO and stored at −20 °C. Ouabain and all chemicals were purchased from Sigma-Aldrich (St. Louis, MO, USA). Cell culture products were purchased from Gibco (Grand Island, NY, USA)

### 4.2. Cell Culture

Porcine kidney proximal tubule cells, LLC-PK1 (ATCC, Manassas, VA, USA), were cultured in Dulbecco’s modified Eagle’s medium (DMEM), supplemented with 10% FBS, NaHCO_3_ (44 mM) and gentamycin (40 mg/L). When cells were about 70–90% confluent, they were washed with phosphate buffered saline (PBS: 125 mM NaCl, 8 mM Na_2_HPO_4_, 2 mM NaH_2_PO_4_, 5 mM KCl) and serum-starved overnight. Then, they were cultured in DMEM supplemented with 2.5% FBS and used for experiments unless stated otherwise.

### 4.3. Cellular Preparation and Treatment with CTS

LLC-PK1 cells grown on 6-well plates were treated with increasing concentrations of CTS for 15 min in serum-free DMEM (time estimated for maximal effect [23,50,97]). The medium was then removed, the cells were washed with PBS and lysed with radioimmunoassay modified buffer (RIPA) containing 1% NP-40, 0.25% deoxycholate, 150 mM NaCl, 1 mM EDTA, 1 mM PMSF, 1 mM Na_3_VO_4_, NaF 1 mM, 10 µg/mL aprotinin, 10 µg/mL leupeptin, 10 nM okadaic acid and 50 mM Tris-HCl (pH 7.4). The cell lysate was centrifuged at 13,000 *g* for 15 min, the supernatant was collected for protein determination and Western blot experiments.

### 4.4. Na^+^/K^+^-ATPase Inhibition Assay

Pig kidney preparation enriched with Na^+^/K^+^-ATPase was kindly provided by Carlos Frederico Leite Fontes (Instituto de Bioquímica Médica, UFRJ, Rio de Janeiro, Brazil) and has been obtained according to the method of Jorgensen [98], with previously described modifications [99]. Ouabain-sensitive ATPase activity was determined by the quantitative determination of inorganic phosphate (Pi) released due to enzymatic hydrolysis of ATP using the colorimetric method of Fiske and Subbarow [100]. For the evaluation of the inhibition of Na^+^/K^+^-ATPase activity, the reaction started with the addition of enzyme preparation in an incubation medium containing 87.6 mM NaCl, 3 mM MgCl_2_, 3 mM ATPNa_2_, 1 mM EGTA, 10 mM NaN_3_, 20 mM Tris-maleate buffer (pH 7.4), with 3 mM KCl (or 1 mM ouabain for basal activity), in the presence of increasing concentrations of CTS, for 1 h at 37 °C [43]. Mean inhibitory concentrations (IC_50_) were estimated fitting the inhibition curves by non-linear regression, as described by Touza et al. [43].

### 4.5. Immunoblot Assay

The technique was performed as described previously [42]. Cellular preparations of LLC-PK1 (20–40 µg protein/well) were separated on 10 or 12% SDS-PAGE gels and electrotransferred to nitrocellulose membranes for 1 h. After protein transfer visualization with Ponceau Red, membranes were incubated for 1 h in 5% skimmed milk dissolved in Tris-buffered saline solution with 0.1% Tween 20 (TBS-T) followed by 1 h incubation with primary (against total and phosphorylated ERK1/2 and GSK-3β protein kinases, 1:1000 dilution, Cell Signaling Technology, Danvers, MA, USA), Bcl-2 and Bax (1:250 dilution, Santa Cruz Biotechnology, Dallas, TX, USA), and secondary peroxidase-conjugated antibodies (rabbit anti-IgG, 1:1000 or 1:4000, Santa Cruz Biotechnology, Dallas, TX, USA). The immunoreactivity was detected by a SuperSignal system (Pierce Biotechnology, Waltham, MA, USA), and the membranes were exposed during 30 s to 5 min to the radiographic film (CL-XPosure, Pierce Biotechnology, Waltham, MA, USA). The autoradiographs were scanned in scanner (HP Scanjet G4050, Hewlett-Packard, Palo Alto, CA, USA) and the quantification was carried out by densitometric analysis using ImageJ software (version 1.42 q, National Institutes of Health, Bethesda, MD, USA). Blots for Bax and Bcl-2, as for total and phosphorylated ERK1/2 and GSK-3β, were stripped and reprobed in the same membrane to ensure reliable comparison.

### 4.6. Cell Counting Assay

In 24-well plates, 1–2 × 10^4^ LLC-PK1 cells/well were treated with 1, 10 or 100 nM CTS for 24, 48, and 72 h. In order to investigate the involvement of intracellular signaling pathways, 100 nM CTS were incubated with or without inhibitors of Src (SU6656, 10 μM; Sigma-Aldrich, St. Louis, MO, USA), ERK1/2-MEK1/2 (U0126, 10 μM; Cell Signaling Technology, Danvers, MA, USA), PI3K (LY294002, 5 μM; Cell Signaling Technology, Danvers, MA, USA), p38 (SB202190, 10 μM; Cell Signaling Technology, Danvers, MA, USA), JNK1/2 (SP600125, 1.5 μM; Cell Signaling Technology, Danvers, MA, USA), or GSK-3β (BIO, 0.5, 1, and 5 μM; Sigma-Aldrich, St. Louis, MO, USA) for 72 h. For MβCD (Sigma-Aldrich, St. Louis, MO, USA) assay, cells were pretreated with 10 mM MβCD for 30 min and then 1 mM MβCD + 100 nM TCB in 2.5% FBS for 72 h. At these time points, two wells from each group were trypsinized and the number of Trypan blue-viable cells was counted in Neubauer chamber (hemocytometer).

### 4.7. MTT Assay

In 96-well plates, 3 × 10^3^ LLC-PK1 cells/well were treated with 100 nM CTS with or without inhibitors of Src (SU6656, 10 μM), ERK1/2-MEK1/2 (U0126, 10 μM), PI3K (LY294002 5 μM), p38 (SB202190, 10 μM), JNK1/2 (SP600125, 1.5 μM), or GSK-3β (BIO, 0.5, 1 and 5 μM) for 72 h. MTT was added directly to culture wells and incubated for 4 h. The absorbance was measured at 570 nm with a 96-well plate reader. The optical density values were normalized to baseline values and presented as percentage of control.

### 4.8. LDH Release

In 24-well plates, 10^4^ LLC-PK1 cells/well were treated with 100 nM CTS for 72 h. Aliquots of 20 μL of media from each well were added to eppendorfs with 1 mL of Tris buffer (pH 7.2) containing NaCl and pyruvate and warmed up to 37 °C. 25 μL NADH solution was added and absorbance was measured at 340 nm in a spectrophotometer at different times (0, 1, 2, and 3 min).

### 4.9. Cell Cycle Analysis

In 24-well plates, 10^4^ LLC-PK1 cells/well were treated with 10 and 100 nM TCB for 48 h. Then, the cells were trypsinized and placed in Falcon tubes. After washing with HBSS (Hank’s Balanced Salt Solution), the cells were incubated with cell-cycle solution containing 50 μg/mL propidium iodide (Sigma-Aldrich, St. Louis, MO, USA), 1 mg/mL RNAse (bovine pancreas, Sigma), and 0.2% Triton X-100, and then cell sorting was performed in a FACSCalibur flow cytometer (BD Biosciences, Franklin Lakes, NJ, USA).

### 4.10. Morphological Analysis by Phase-Contrast and Fluorescence Microscopy

In 24-well plates, 5 × 10^3^ LLC-PK1 cells/well were treated with 100 nM TCB for 24 h. For BAPTA-AM assay, cells were pretreated with 5 μM BAPTA-AM for 30 min and then treated with TCB. For nuclear fluorescence staining, cells were cultured in coverslips. After 24 h, they were washed with PBS, fixed with 4% paraformaldehyde for 10 min, washed again and then incubated with 0.2% Triton X-100 for 5 min. Cells were incubated with Hoechst 33,342 (1:10,000 dilution, Thermo Fisher Scientific, Waltham, MA, USA) for 30 min. Cells were examined and photographed by phase-contrast and fluorescence microscopy (Olympus CellSens Software 1.5, IX71 Olympus Microscope, Center Valley, PA, USA).

### 4.11. Dual Luciferase Reporter Assay

LLC-PK1 cell lines were transfected using Lipofectamine 3000 Transfection Reagent (Invitrogen, Carlsbad, CA, USA) and performed in triplicate. Cells were plated into 96-well plates and transfected in the following day with 100 ng of TOPFLASH plasmid, 10 ng of CMV promoter *Renilla* plasmid and 100 ng of each experimental plasmid. Cells were treated for 24 h with TCB or vehicle, with or without Wnt3aCM 24 h post-transfection. Wnt3a CM and L-cell CM were obtained according to ATCC protocol. Cells were lysed with passive lysis buffer (Promega, Madison, WI, USA) and TOPFLASH reporter activity was measured according to the manufacturer protocol (Dual-Luciferase Reporter Assay System, Promega, Madison, WI, USA). The triplicate assays data were normalized to the empty vector control.

### 4.12. Statistical Analysis

Statistical analyzes were performed by analysis of variance—one-way or two-way (for cell counting in Figure 3 and Figure S3) ANOVA—followed by Dunnett test using GraphPad Prism^®^ software (version 6, GraphPad Software, La Jolla, CA, USA). Values of *p* < 0.05 were considered statistically significant. The data represent the results of three or more experiments and were expressed as mean ± standard error of the mean (SEM).

## 5. Conclusions

In conclusion, the present study strongly suggests that structurally similar bufadienolides—marinobufagin and telocinobufagin—present functional selectivity promoting divergent effects by different mechanisms of action despite acting on the same receptor (Na^+^/K^+^-ATPase) and contribute to the understanding of the existence of different endogenous CTS in mammals.

## Supplementary Materials

Supplementary Materials can be found at http://www.mdpi.com/1422-0067/19/9/2769/s1.

## References

[1] Liu J., Xie Z.J. (2010). The sodium pump and cardiotonic steroids-induced signal transduction protein kinases and calcium-signaling microdomain in regulation of transporter trafficking. Biochim. Biophys. Acta.

[2] Pierre S.V., Xie Z.J. (2006). The Na,K-ATPase receptor complex: Its organization and membership. Cell Biochem. Biophys..

[3] Li Z., Xie Z. (2009). The Na/K-ATPase/Src complex and cardiotonic steroid-activated protein kinase cascades. Eur. J. Physiol..

[4] Bagrov A.Y., Shapiro J.I., Fedorova O.V. (2009). Endogenous cardiotonic steroids: Physiology, pharmacology, and novel therapeutic targets. Pharmacol. Rev..

[5] Ferrandi M., Manunta P., Ferrari P., Bianchi G. (2005). The endogenous ouabain: Molecular basis of its role in hypertension and cardiovascular complications. Front. Biosci..

[6] Nesher M., Shpolansky U., Rosen H., Lichtstein D. (2007). The digitalis-like steroid hormones: New mechanisms of action and biological significance. Life Sci..

[7] Puschett J.B., Agunanne E., Uddin M.N. (2010). Emerging role of the bufadienolides in cardiovascular and kidney diseases. Am. J. Kidney Dis..

[8] Prassas I., Diamandis E.P. (2008). Novel therapeutic applications of cardiac glycosides. Nat. Rev. Drug Discov..

[9] Sousa L.Q., Machado K.D., Oliveira S.F., Araújo L.D., Monção-Filho E.D., Melo-Cavalcante A.A., Vieira-Júnior G.M., Ferreira P.M. (2017). Bufadienolides from amphibians: A promising source of anticancer prototypes for radical innovation, apoptosis triggering and Na^+^/K^+^-ATPase inhibition. Toxicon.

[10] Mijatovic T., Van Quaquebeke E., Delest B., Debeir O., Darro F., Kiss R. (2007). Cardiotonic steroids on the road to anti-cancer therapy. Biochim. Biophys. Acta.

[11] Slingerland M., Cerella C., Guchelaar H.J., Diederich M., Gelderblom H. (2013). Cardiac glycosides in cancer therapy: From preclinical investigations towards clinical trials. Investig. New Drugs.

[12] Gonçalves-de-Albuquerque C.F., Ribeiro Silva A., Ignácio da Silva C., Castro-Faria-Neto H.C., Burth P. (2017). Na/K-Pump and Beyond: Na/K-ATPase as a Modulator of Apoptosis and Autophagy. Molecules.

[13] McConkey D.J., Lin Y., Nutt L.K., Ozel H.Z., Newman R.A. (2000). Cardiac glycosides stimulate Ca^2+^ increases and apoptosis in androgen-independent, metastatic human prostate adenocarcinoma cells. Cancer Res..

[14] Tian J., Li X., Liang M., Liu L., Xie J.X., Ye Q., Kometiani P., Tillekeratne M., Jin R., Xie Z. (2009). Changes in sodium pump expression dictate the effects of ouabain on cell growth. J. Biol. Chem..

[15] Nesher M., Shpolansky U., Viola N., Dvela M., Buzaglo N., Cohen Ben-Ami H., Rosen H., Lichtstein D. (2010). Ouabain attenuates cardiotoxicity induced by other cardiac steroids. Br. J. Pharmacol..

[16] Manunta P., Hamilton J., Rogowski A.C., Hamilton B.P., Hamlyn J.M. (2000). Chronic hypertension induced by ouabain but not digoxin in the rat: Antihypertensive effect of digoxin and digitoxin. Hypertens. Res..

[17] Zulian A., Linde C.L., Pulina M.V., Baryshnikov S.G., Papparella I., Hamlyn J.M., Golovina V.A. (2013). Activation of c-SRC underlies the differential effects of ouabain and digoxin on Ca^2+^ signaling in arterial smooth muscle cells. Am. J. Physiol. Cell Physiol..

[18] Akimova O.A., Bagrov A.Y., Lopina O.D., Kamernitsky A.V., Tremblay J., Hamet P., Orlov S.N. (2005). Cardiotonic steroids differentially affect intracellular Na^+^ and [Na^+^]_i_/[K^+^]_i_-independent signaling in C7-MDCK cells. J. Biol. Chem..

[19] Arnaud-Batista F.J., Costa G.T., Oliveira I.M., Costa P.P., Santos C.F., Fonteles M.C., Uchôa D.E., Silveira E.R., Cardi B.A., Carvalho K.M. (2012). Natriuretic effect of bufalin in isolated rat kidneys involves activation of the Na^+^-K^+^-ATPase-Src kinase pathway. Am. J. Physiol. Renal Physiol..

[20] Galandrin S., Oligny-Longpré G., Bouvier M. (2007). The evasive nature of drug efficacy: Implications for drug discovery. Trends Pharmacol. Sci..

[21] Urban J.D., Clarke W.P., Von Zastrow M., Nichols D.E., Kobilka B., Weinstein H., Javitch J.A., Roth B.L., Christopoulos A., Sexton P.M. (2007). Functional selectivity and classical concepts of quantitative pharmacology. J. Pharmacol. Exp. Ther..

[22] Kenakin T. (2011). Functional selectivity and biased receptor signaling. J. Pharmacol. Exp. Ther..

[23] Quintas L.E.M., Pierre S.V., Liu L., Bai Y., Liu X., Xie Z.J. (2010). Alterations of Na^+^/K^+^-ATPase function in caveolin-1 knockout cardiac fibroblasts. J. Mol. Cell. Cardiol..

[24] Aydemir-Koksoy A., Abramowitz J., Allen J. (2001). Ouabain-induced signaling and vascular smooth muscle cell proliferation. J. Biol. Chem..

[25] Abramowitz J., Dai C., Hirschi K.K., Dmitrieva R.I., Doris P.A., Liu L., Allen J.C. (2003). Ouabain- and marinobufagenin-induced proliferation of human umbilical vein smooth muscle cells and rat vascular smooth muscle cell line, A7r5. Circulation.

[26] Yuan Z., Cai T., Tian J., Ivanov A.V., Giovannucci D.R., Xie Z. (2005). Na/K-ATPase tethers phospholipase C and IP3 receptor into a calcium-regulatory complex. Mol. Biol. Cell..

[27] Harr M.W., Distelhorst C.W. (2010). Apoptosis and autophagy: Decoding calcium signals that mediate life or death. Cold Spring Harb. Perspect. Biol..

[28] Liu L., Mohammadi K., Aynafshar B., Wang H., Li D., Liu J., Ivanov A.V., Xie Z., Askari A. (2003). Role of caveolae in signal-transducing function of cardiac Na^+^/K^+^-ATPase. Am. J. Physiol. Cell Physiol..

[29] Bai Y., Wu J., Li D., Morgan E.E., Liu J., Zhao X., Walsh A., Saikumar J., Tinkel J., Joe B. (2016). Differential roles of caveolin-1 in ouabain-induced Na^+^/K^+^-ATPase cardiac signaling and contractility. Physiol. Genom..

[30] Wang H., Haas M., Liang M., Cai T., Tian J., Li S., Xie Z. (2004). Ouabain assembles signaling cascades through the caveolar Na^+^/K^+^-ATPase. J. Biol. Chem..

[31] Nguyen A.N., Jansson K., Sánchez G., Sharma M., Reif G.A., Wallace D.P., Blanco G. (2011). Ouabain activates the Na-K-ATPase signalosome to induce autosomal dominant polycystic kidney disease cell proliferation. Am. J. Physiol. Renal Physiol..

[32] Oliveira T.N., Possidonio A.C., Soares C.P., Ayres R., Costa M.L., Quintas L.E.M., Mermelstein C. (2015). The role of Na^+^/K^+^-ATPase during chick skeletal myogenesis. PLoS ONE.

[33] Afroze S.H., Sloan J., Osuji G.C., Drever N., Pilkinton K., Zawieja D.C., Kuehl T.J., Uddin M.N. (2016). Cinobufotalin impedes Sw.71 cytotrophoblast cell line function via cell cycle arrest and apoptotic signaling. Mol. Cell. Biochem..

[34] Kometiani P., Liu L., Askari A. (2005). Digitalis-induced signaling by Na^+^/K^+^-ATPase in human breast cancer cells. Mol. Pharmacol..

[35] Deng L.J., Hu L.P., Peng Q.L., Yang X.L., Bai L.L., Yiu A., Li Y., Tian H.Y., Ye W.C., Zhang D.M. (2014). Hellebrigenin induces cell cycle arrest and apoptosis in human hepatocellular carcinoma HepG2 cells through inhibition of Akt. Chem. Biol. Interact..

[36] Wang H., Zhang C., Xu L., Zang K., Ning Z., Jiang F., Chi H., Zhu X., Meng Z. (2016). Bufalin suppresses hepatocellular carcinoma invasion and metastasis by targeting HIF-1α via the PI3K/AKT/mTOR pathway. Oncotarget.

[37] Zhang G., Wang C., Sun M., Li J., Wang B., Jin C., Hua P., Song G., Zhang Y., Nguyen L.L. (2016). Cinobufagin inhibits tumor growth by inducing intrinsic apoptosis through AKT signaling pathway in human nonsmall cell lung cancer cells. Oncotarget.

[38] McCubrey J.A., Fitzgerald T.L., Yang L.V., Lertpiriyapong K., Steelman L.S., Abrams S.L., Montalto G., Cervello M., Neri L.M., Cocco L. (2017). Roles of GSK-3 and microRNAs on epithelial mesenchymal transition and cancer stem cells. Oncotarget.

[39] Yin P.H., Liu X., Qiu Y.Y., Cai J.F., Qin J.M., Zhu H.R., Li Q. (2012). Anti-tumor activity and apoptosis-regulation mechanisms of bufalin in various cancers: New hope for cancer patients. Asian Pac. J. Cancer Prev..

[40] Blanco G., Mercer R.W. (1998). Isozymes of the Na-K-ATPase: Heterogeneity in structure, diversity in function. Am. J. Physiol. Renal Physiol..

[41] Gable M.E., Ellis L., Fedorova O.V., Bagrov A.Y., Askari A. (2017). Comparison of digitalis sensitivities of Na^+^/K^+^-ATPases from human and pig kidneys. ACS Omega.

[42] Godinho A.N., Costa G.T., Oliveira N.O., Cardi B.A., Uchoa D.E.A., Silveira E.R., Quintas L.E.M., Noël F., Fonteles M.C., Carvalho K.M. (2017). Effects of cardiotonic steroids on isolated perfused kidney and NHE3 activity in renal proximal tubules. Biochim. Biophys. Acta.

[43] Touza N.A., Pôças E.S., Quintas L.E.M., Cunha-Filho G., Santos M.L., Noël F. (2011). Inhibitory effect of combinations of digoxin and endogenous cardiotonic steroids on Na^+^/K^+^-ATPase activity in human kidney membrane preparation. Life Sci..

[44] Xie Z., Cai T. (2003). Na^+^-K^+^-ATPase-mediated signal transduction: From protein interaction to cellular function. Mol. Interv..

[45] Cui X., Xie Z. (2017). Protein interaction and Na/K-ATPase-mediated signal transduction. Molecules.

[46] Trevisi L., Visentin B., Cusinato F., Pighin I., Luciani S. (2004). Antiapoptotic effect of ouabain on human umbilical vein endothelial cells. Biochem. Biophys. Res. Commun..

[47] Khundmiri S.J., Amin V., Henson J., Lewis J., Ameen M., Rane M.J., Delamere N.A. (2007). Ouabain stimulates protein kinase B (Akt) phosphorylation in opossum kidney proximal tubule cells through an ERK-dependent pathway. Am. J. Physiol. Cell Physiol..

[48] Nguyen A.N., Wallace D.P., Blanco G. (2007). Ouabain binds with high affinity to the Na,K-ATPase in human polycystic kidney cells and induces extracellular signal-regulated kinase activation and cell proliferation. J. Am. Soc. Nephrol..

[49] Silva E., Soares-da-Silva P. (2011). Long-term regulation of Na^+^,K^+^-ATPase in opossum kidney cells by ouabain. J. Cell Physiol..

[50] Lucas T.F., Amaral L.S., Porto C.S., Quintas L.E.M. (2012). Na^+^/K^+^-ATPase α1 isoform mediates ouabain-induced expression of cyclin D1 and proliferation of rat sertoli cells. Reproduction.

[51] Haas M., Askari A., Xie Z. (2000). Involvement of Src and epidermal growth factor receptor in the signal-transducing function of Na^+^/K^+^-ATPase. J. Biol. Chem..

[52] Liang M., Cai T., Tian J., Qu W., Xie Z.J. (2006). Functional characterization of Src-interacting Na/K-ATPase using RNA interference assay. J. Biol. Chem..

[53] Sayed M., Drummond C.A., Evans K.L., Haller S.T., Liu J., Xie Z., Tian J. (2014). Effects of Na/K-ATPase and its ligands on bone marrow stromal cell differentiation. Stem Cell Res..

[54] Dmitrieva R.I., Doris P.A. (2003). Ouabain is a potent promoter of growth and activator of ERK1/2 in ouabain-resistant rat renal epithelial cells. J. Biol. Chem..

[55] Kulikov A., Eva A., Kirch U., Boldyrev A., Scheiner-Bobis G. (2007). Ouabain activates signaling pathways associated with cell death in human neuroblastoma. Biochim. Biophys. Acta.

[56] Karpova L.V., Bulygina E.R., Boldyrev A.A. (2010). Different neuronal Na^+^/K^+^-ATPase isoforms are involved in diverse signaling pathways. Cell Biochem. Funct..

[57] Chueh S., Guh J., Chen J., Lai M., Teng C. (2001). Dual effects of ouabain on the regulation of proliferation and apoptosis in human prostatic smooth muscle cells. J. Urol..

[58] Li M., Wang Q., Guan L. (2007). Effects of ouabain on proliferation intracellular free calcium and c-myc mRNA expression in vascular smooth muscle cells. J. Comp. Physiol. B.

[59] Olej B., dos Santos N.F., Leal L., Rumjanek V.M. (1998). Ouabain induces apoptosis on PHA-activated lymphocytes. Biosci. Rep..

[60] Pchejetski D., Taurin S., Der Sarkissian S., Lopina O.D., Pshezhetsky A.V., Tremblay J., deBlois D., Hamet P., Orlov S.N. (2003). Inhibition of Na^+^,K^+^-ATPase by ouabain triggers epithelial cell death independently of inversion of the [Na^+^]_i_/[K^+^]_i_ ratio. Biochem. Biophys. Res. Commun..

[61] Orlov S.N., Thorin-Trescases N., Pchejetski D., Taurin S., Farhat N., Tremblay J., Thorin E., Hamet P. (2004). Na^+^/K^+^ pump and endothelial cell survival: [Na^+^]_i_/[K^+^]_i_-independent necrosis triggered by ouabain, and protection against apoptosis mediated by elevation of [Na^+^]_i_. Pflugers Arch..

[62] Winnicka K., Bielawski K., Bielawska A., Miltyk W. (2010). Dual effects of ouabain, digoxin and proscillaridin A on the regulation of apoptosis in human fibroblasts. Nat. Prod. Res..

[63] Mijatovic T., Dufrasne F., Kiss R. (2012). Cardiotonic steroids-mediated targeting of the Na^+^/K^+^-ATPase to combat chemoresistant cancers. Curr. Med. Chem..

[64] Akera T., Brody T.M. (1978). The role of Na^+^,K^+^-ATPase in the inotropic action of digitalis. Pharmacol. Rev..

[65] Lin H., Juang J.L., Wang P.S. (2004). Involvement of Cdk5/p25 in digoxin-triggered prostate cancer cell apoptosis. J. Biol. Chem..

[66] Yeh J.Y., Huang W.J., Kan S.F., Wang P.S. (2003). Effects of bufalin and cinobufagin on the proliferation of androgen dependent and independent prostate cancer cells. Prostate.

[67] Cai H., Wu L., Qu W., Malhotra D., Xie Z., Shapiro J.I., Liu J. (2008). Regulation of apical NHE3 trafficking by ouabain-induced activation of the basolateral Na^+^-K^+^-ATPase receptor complex. Am. J. Physiol. Cell Physiol..

[68] Liang M., Tian J., Liu L., Pierre S., Liu J., Shapiro J., Xie Z.J. (2007). Identification of a pool of non-pumping Na/K-ATPase. J. Biol. Chem..

[69] Cerella C., Dicato M., Diederich M. (2013). Assembling the puzzle of anti-cancer mechanisms triggered by cardiac glycosides. Mitochondrion.

[70] Akimova O.A., Lopina O.D., Rubtsov A.M., Gekle M., Tremblay J., Hamet P., Orlov S.N. (2009). Death of ouabain-treated renal epithelial cells: Evidence for p38 MAPK-mediated Na_i_^+^/K_i_^+^-independent signaling. Apoptosis.

[71] Akimova O.A., Lopina O.D., Rubtsov A.M., Hamet P., Orlov S.N. (2010). Investigation of mechanism of p38 MAPK activation in renal epithelial cell from distal tubules triggered by cardiotonic steroids. Biochemistry.

[72] Ehrig J.C., Afroze S.H., Reyes M., Allen S.R., Drever N.S., Pilkinton K.A., Kuehl T.J., Uddin M.N. (2015). A p38 mitogen-activated protein kinase inhibitor attenuates cardiotonic steroids-induced apoptotic and stress signaling in a Sw-71 cytotrophoblast cell line. Placenta.

[73] Xie C.M., Chan W.Y., Yu S., Zhao J., Cheng C.H. (2011). Bufalin induces autophagy-mediated cell death in human colon cancer cells through reactive oxygen species generation and JNK activation. Free Radic. Biol. Med..

[74] Trenti A., Grumati P., Cusinato F., Orso G., Bonaldo P., Trevisi L. (2014). Cardiac glycoside ouabain induces autophagic cell death in non-small cell lung cancer cells via a JNK-dependent decrease of Bcl-2. Biochem. Pharmacol..

[75] Zhao H., Li Q., Pang J., Jin H., Li H., Yang X. (2017). Blocking autophagy enhances the pro-apoptotic effect of bufalin on human gastric cancer cells through endoplasmic reticulum stress. Biol. Open.

[76] Beurel E., Jope R.S. (2006). The paradoxical pro- and anti-apoptotic actions of GSK3 in the intrinsic and extrinsic apoptosis signaling pathways. Prog. Neurobiol..

[77] Kotova O., Al-Khalili L., Talia S., Hooke C., Fedorova O.V., Bagrov A.Y., Chibalin A.V. (2006). Cardiotonic steroids stimulate glycogen synthesis in human skeletal muscle cells via a Src- and ERK1/2-dependent mechanism. J. Biol. Chem..

[78] Gai J.Q., Sheng X., Qin J.M., Sun K., Zhao W., Ni L. (2016). The effect and mechanism of bufalin on regulating hepatocellular carcinoma cell invasion and metastasis via Wnt/β-catenin signaling pathway. Int. J. Oncol..

[79] Ichikawa M., Sowa Y., Iizumi Y., Aono Y., Sakai T. (2015). Resibufogenin induces G1-phase arrest through the proteasomal degradation of cyclin D1 in human malignant tumor cells. PLoS ONE.

[80] Kang X.H., Zhang J.H., Zhang Q.Q., Cui Y.H., Wang Y., Kou W.Z., Miao Z.H., Lu P., Wang L.F., Xu Z.Y. (2017). Degradation of Mcl-1 through GSK-3β activation regulates apoptosis induced by bufalin in non-small cell lung cancer H1975 cells. Cell Physiol. Biochem..

[81] Sengupta P.K., Bouchie M.P., Kukuruzinska M.A. (2010). N-Glycosylation gene DPAGT1 is a target of the Wnt/β-catenin signaling pathway. J. Biol. Chem..

[82] Yamamoto H., Awada C., Matsumoto S., Kaneiwa T., Sugimoto T., Takao T., Kikuchi A. (2015). Basolateral secretion of Wnt5a in polarized epithelial cells is required for apical lumen formation. J. Cell Sci..

[83] Ma Y., Zhu B., Liu X., Yu H., Yong L., Liu X., Shao J., Liu Z. (2015). Inhibition of oleandrin on the proliferation show and invasion of osteosarcoma cells in vitro by suppressing Wnt/β-catenin signaling pathway. J. Exp. Clin. Cancer Res..

[84] Shen Y., Wang Q., Tian Y. (2016). Reversal effect of ouabain on multidrug resistance in esophageal carcinoma EC109/CDDP cells by inhibiting the translocation of Wnt/β-catenin into the nucleus. Tumour Biol..

[85] Fedorova L.V., Raju V., El-Okdi N., Shidyak A., Kennedy D.J., Vetteth S., Giovannucci D.R., Bagrov A.Y., Fedorova O.V., Shapiro J.I. (2009). The cardiotonic steroid hormone marinobufagenin induces renal fibrosis: Implication of epithelial-to-mesenchymal transition. Am. J. Physiol. Renal Physiol..

[86] Venugopal J., McDermott J., Sanchez G., Sharma M., Barbosa L., Reif G.A., Wallace D.P., Blanco G. (2017). Ouabain promotes partial epithelial to mesenchymal transition (EMT) changes in human autosomal dominant polycystic kidney disease (ADPKD) cells. Exp. Cell Res..

[87] Larre I., Ponce A., Fiorentino R., Shoshani L., Contreras R.G., Cereijido M. (2006). Contacts and cooperation between cells depend on the hormone ouabain. Proc. Natl. Acad. Sci. USA.

[88] Kenakin T., Williams M. (2014). Defining and characterizing drug/compound function. Biochem. Pharmacol..

[89] Runge T.M., Stephens J.C., Holden P., Havemann D.F., Kilgore W.M., Dale E.M., Dalton R.E. (1975). Pharmacodynamic distinctions between ouabain, digoxin and digitoxin. Arch. Int. Pharmacodyn. Ther..

[90] Joubert P.H. (1990). Are all cardiac glycosides pharmacodynamically similar?. Eur. J. Clin. Pharmacol..

[91] Pamnani M.B., Chen S., Bryant H.J., Schooley J.F., Eliades D.C., Yuan C.M., Haddy F.J. (1991). Effects of three sodium-potassium adenosine triphosphatase inhibitors. Hypertension.

[92] Laursen M., Gregersen J.L., Yatime L., Nissen P., Fedosova N.U. (2015). Structures and characterization of digoxin- and bufalin-bound Na^+^,K^+^-ATPase compared with the ouabain-bound complex. Proc. Natl. Acad. Sci. USA.

[93] Klimanova E.A., Petrushanko I.Y., Mitkevich V.A., Anashkina A.A., Orlov S.N., Makarov A.A., Lopina O.D. (2015). Binding of ouabain and marinobufagenin leads to different structural changes in Na,K-ATPase and depends on the enzyme conformation. FEBS Lett..

[94] Song H., Karashima E., Hamlyn J.M., Blaustein M.P. (2014). Ouabain-digoxin antagonism in rat arteries and neurones. J. Physiol..

[95] Feldmann T., Glukmann V., Medvenev E., Shpolansky U., Galili D., Lichtstein D., Rosen H. (2007). Role of endosomal Na^+^-K^+^-ATPase and cardiac steroids in the regulation of endocytosis. Am. J. Physiol. Cell Physiol..

[96] Cunha-Filho G.A., Resck I.S., Cavalcanti B.C., Pessoa C.O., Moraes M.O., Ferreira J.R., Rodrigues F.A., Dos Santos M.L. (2010). Cytotoxic profile of natural and some modified bufadienolides from toad *Rhinella schneideri* parotoid gland secretion. Toxicon.

[97] Pierre S.V., Sottejeau Y., Gourbeau J.M., Sánchez G., Shidyak A., Blanco G. (2008). Isoform specificity of Na-K-ATPase-mediated ouabain signaling. Am. J. Physiol. Renal Physiol..

[98] Jorgensen P.L. (1975). Techniques for the study of steroid effects on membraneous (Na^+^ + K^+^)-ATPase. Methods Enzymol..

[99] Fontes C.F., Lopes F.E., Scofano H.M., Barrabin H., Norby J.G. (1999). Stimulation of ouabain binding to Na,K-ATPase in 40% dimethyl sulfoxide by a factor from Na,K-ATPase preparations. Arch. Biochem. Biophys..

[100] Fiske C.H., Subbarow Y. (1925). The colorimetric determination of phosphorus. J. Biol. Chem..

