# Supplementary material for: Telocinobufagin and Marinobufagin Produce Different Effects in LLC-PK1 Cells: A Case of Functional Selectivity of Bufadienolides
Source: Int J Mol Sci. 2018 Sep 14;19(9):2769. doi: 10.3390/ijms19092769 (PMC6163863; doi:10.3390/ijms19092769)
Supplement: Supplementary file 1 [file ijms-19-02769-s001.pdf]

International Journal of
Molecular Sciences

MDPI

Article

Telocinobufagin and Marinobufagin Produce Different Effects in LLC-PK1 Cells: A Case of Functional Selectivity of Bufadienolides

Luciana S. Amaral [1], Jainne Martins Ferreira [1], Danilo Predes [1], José Garcia Abreu [1], François Noël [1] and Luis Eduardo M. Quintas [1,*]

[1] Institute of Biomedical Sciences, Federal University of Rio de Janeiro, Rio de Janeiro, Brazil
* Correspondence: lquintas@icb.ufrj.br; Tel.: +55-21-2562-6732

Supplementary Information

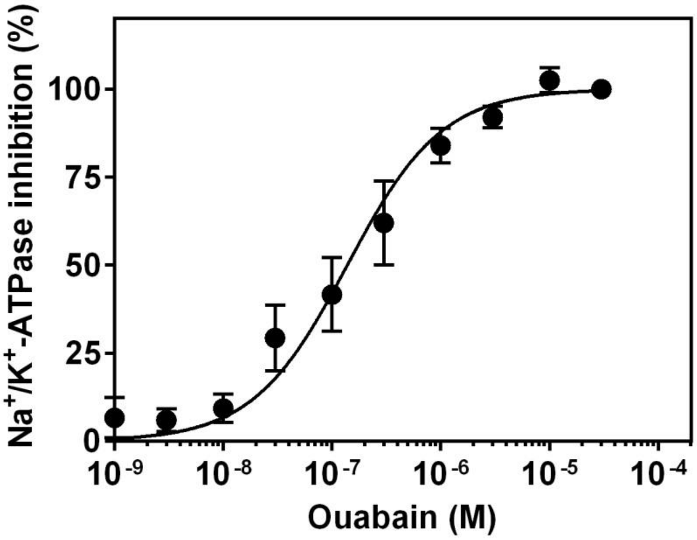

Figure 1S. Inhibition curve of pig kidney Na^+/K^+-ATPase activity by ouabain (OUA). Preparation of Na^+/K^+-ATPase from pig kidney was incubated with increasing concentrations of ouabain for 1 h. Each point represents the mean ± SEM of 3 experiments performed in triplicate.

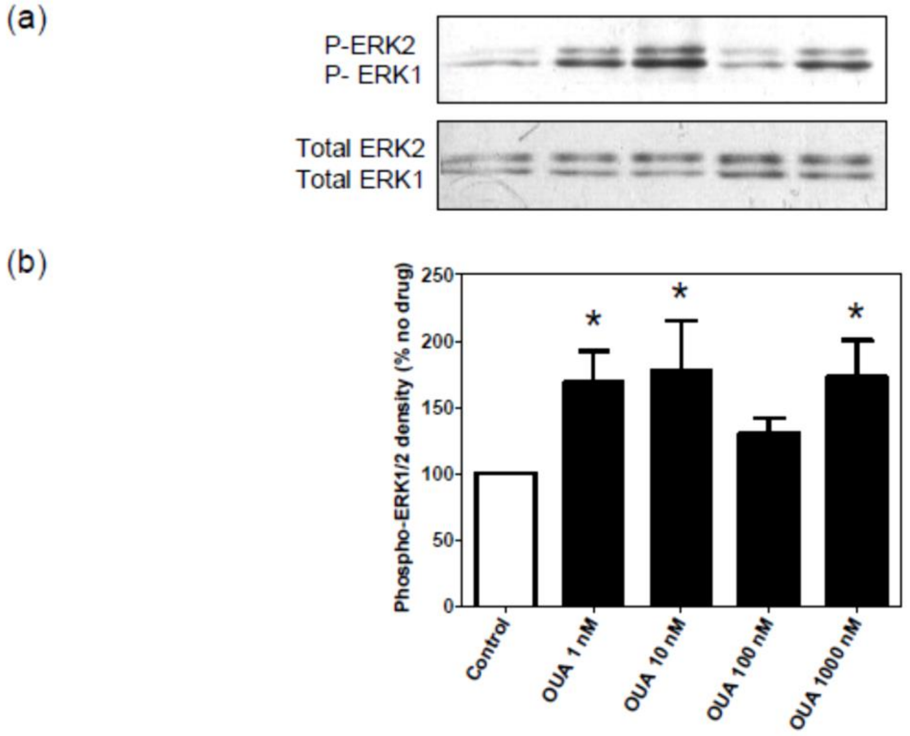

Figure 2S. ERK1/2 activation by different concentrations of ouabain (OUA). Serum-starved LLC-PK1 cells were treated with 1, 10, 100 or 1000 nM OUA for 15 min. Representative Western blots of phospho- and total ERK1/2 for OUA (a) and relative optical density quantification (b). Data are the mean ± SEM of at least 3 independent experiments. * $p < 0.05$ vs control.

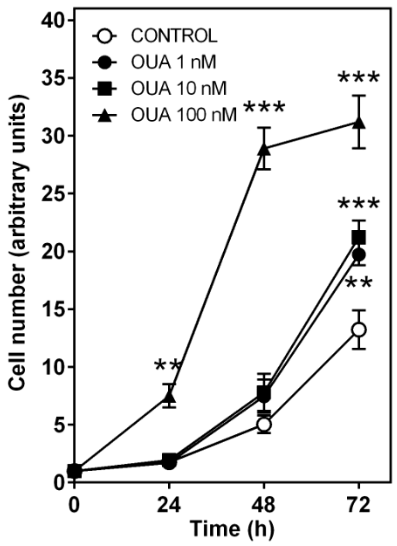

Figure 3S. Cell proliferation of LLC-PK1 cells treated with ouabain (OUA). Serum-starved LLC-PK1 cells were treated with 1, 10 and 100 nM OUA in 2.5% FBS for 24, 48 and 72 h, and then Trypan blue-free viable cells were counted in Neubauer chamber. Each point represents the mean ± SEM of 3 independent experiments performed in duplicate. ** $p < 0.01$; *** $p < 0.005$ vs control.

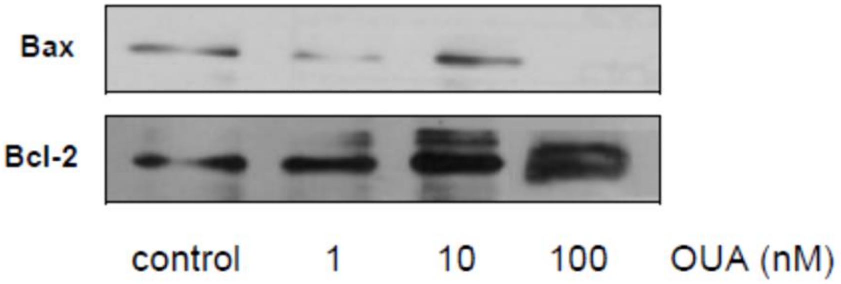

Figure 4S. Bax and Bcl-2 expression in LLC-PK1 cells treated with ouabain (OUA). Serum-starved LLC-PK1 cells were treated with 1, 10 and 100 nM OUA in 2.5% FBS for 72 h. Representative Western blots of the pro-apoptotic Bax and anti-apoptotic Bcl-2 for OUA. Similar results were observed in 2 independent experiments.

© 2018 by the authors. Licensee MDPI, Basel, Switzerland. This article is an open access article distributed under the terms and conditions of the Creative Commons Attribution (CC BY) license (http://creativecommons.org/licenses/by/4.0/).
